# Cadmium and polyvinyl chloride microplastics induce mitochondrial damage and apoptosis under oxidative stress in duck kidney

**DOI:** 10.1016/j.psj.2024.104490

**Published:** 2024-11-12

**Authors:** Hui Zou, Jie Song, Xianzu Luo, Waseem Ali, Sifan Li, Ling Xiong, Yan Chen, Yan Yuan, Yonggang Ma, Xishuai Tong, Zongping Liu

**Affiliations:** a College of Veterinary Medicine, Yangzhou University, Yangzhou, Jiangsu 225009, PR China; b Joint International Research Laboratory of Agriculture and Agri-Product Safety of the Ministry of Education of China, Yangzhou University, Yangzhou, Jiangsu 225009, PR China; c Agri-Product Safety of the Ministry of Education of China, Yangzhou University, Yangzhou, Jiangsu 225009, PR China; d Jiangsu Co-innovation Center for Prevention and Control of Important Animal Infectious Diseases and Zoonoses, Yangzhou, Jiangsu 225009, PR China; e Institutes of Agricultural Science and Technology Development, Yangzhou University, Yangzhou, Jiangsu 225009, PR China

**Keywords:** Apoptosis, Cadmium, Duck kidney, Oxidative stress, PVC-MPs

## Abstract

Polyvinyl chloride microplastics **(PVC-MPs)** and Cadmium **(Cd)** are widely occurring water pollutants that interact with each other to exert toxic effects. As a waterfowl, Muscovy duck is more susceptible to PVC-MPs and Cd than land poultry. In this study, Muscovy duck was used as a research model, and 10 mg/L PVC-MPs and 50 mg/kg Cd were used alone and in combine to explore the effect on the kidney of Muscovy duck. We found that treatment of Cd or PVC-MPs alone changed the kidney weight, increased creatinine and urea nitrogen content, and disrupted oxidative balance and macro/trace element metabolism, while the combination of PVC-MPs+Cd reduced the accumulation of Cd in the kidney. In addition, treatment of Cd and PVC-MPs alone caused mitochondrial damage, increase or decrease of mitochondria-associated proteins (Fis1, Drp1, PGC-1α, Nrf1), and Nrf2 signaling pathway plays a key role in detoxification and alleviation of oxidative stress, and we found that PVC-MPs+Cd treatment recovered related proteins (Nrf2, Keap-1, HO-1, NQO1, AC-SOD_2_, SOD_2_) compared with the Cd and PVC-MPs alone treatment. Finally, we detected changes in apoptosis-related proteins and genes (Caspase-3, Caspase-9, Bax, Bcl-2, Cytc) and TUNEL staining, and after PVC-MPs+Cd treatment, apoptosis-related proteins/genes recovered and the apoptosis rate decreased compared with the Cd and PVC-MPs alone treatment. These results indicate that renal function is impaired, oxidative stress and trace element metabolism disorder, nuclear factor-E2 related factor 2 (Nrf2) is activated into the nucleus to induce the expression of related antioxidant proteins (such as HO-1, NQO1). These injuries can induce mitochondrial damage and eventually lead to renal cell apoptosis. To sum up, these evidence show that Cd or PVC-MPs can induce kidney oxidative damage, trace element metabolism disorder, mitochondrial damage and apoptosis.

## Introduction

Microplastics were first reported in 2004 (Thompson et al., 2004). Recent data shows that only 9% of the world's plastic waste is recycled, 19% is incinerated, nearly 50% is stored in sanitary landfills, and the remaining 22% is poorly managed plastic waste that flows into the environment in various ways (OECD, 2022). PVC-MPs, due to their widespread use, are found in various environments around the world, including Austria, India, the Arctic, and Antarctica (Kau et al., 2024; Materić et al., 2022; Nandi et al., 2024). At present, we have a relatively clear understanding of the way MPs damage the body, and the damage can be summarized into two categories, one is physical damage, and the other is toxicological damage. Physical injuries such as intestinal obstruction, ulcers, and intestinal perforation, as well as pseudo satiety, are the main ones (Derraik, 2002), toxicological impairment is primarily due to reproductive dysfunction, activation of inflammatory responses, and immunodeficiency, both of which may lead to increased mortality (Carbery et al., 2018; Fossi et al., 2018; Roman et al., 2019). At present, there is a large amount of data to prove that ingestion of microplastics can induce oxidative stress and functional damage in organs such as the gastrointestinal tract, liver, kidneys, and lungs, and reduce their survival rate (Ding et al., 2024; Farag et al., 2024; Jin et al., 2024; Li et al., 2024). In addition, some microplastic particles can be deposited directly into lakes and rivers through air deposition, which can pollute urban water sources (Sun et al., 2022). In this context, aquatic organisms are particularly susceptible to the adverse effects of microplastics particles. As early as 2018, a study found that MPs were found in the feathers of geese and ducks, and there was a positive correlation with MPs in feces (Reynolds and Ryan, 2018), Haave et al. (2021) used pyrolysis-gas chromatography-mass spectrometry **(Py-GC/MS)** to examine the lung organs of three seabirds off the west coast of Norway and found the presence of MPS. These results fully illustrate the aggregation of MPs in various tissues of the body.

A series of industrial activities, such as mining and smelting, result in the release of large amounts of Cd into the environment (Kubier and Pichler, 2019). Long ago, the US Agency for Poisons and Disease Registry **(ATSDR)** lists Cd as the sixth most toxic substance threatening human health (Lichtveld et al., 1992), and the International Agency for Research on Cancer **(IARC)** also lists Cd as a Class IA carcinogen (Rindi et al., 2018). Cd diffuses in the environment through several carbon processes (e.g., mining, smelting, nickel/ Cd batteries, pigments, plastic stabilizers, phosphate fertilizers, and composting) (Nordberg et al., 2018). On the one hand, like all other metals, Cd remains in the environment indefinitely and increases the amount of Cd in the environment over time due to its non-biodegradability. On the other hand, Cd and Cu have a higher transfer quotient (Cd>Cu>Pb>Fe) compared to Pb and Fe (Khan et al., 2008), which leads to disturbance of the metabolic balance of trace elements and disruption of the normal structure and function of the cell. Exposure to Cd in the general population may be due to ingestion of contaminated food or water and tobacco smoke or air inhalation of particles (Souza-Arroyo et al., 2022). According to recent population monitoring and dietary assessment reports, long-term exposure to low levels of environmental Cd is inevitable (Satarug and Moore, 2004). Because diet accounts for about 90% of Cd exposure, in addition to occupational exposure and personal tobacco consumption (Satarug et al., 2022).

In recent decades, with the advancement of China's industrialization, a large amount of wastewater containing heavy metals **(HMW)** has been discharged, causing serious environmental problems (Chen et al., 2021; Qin et al., 2021). Many farmlands, wetlands, forests and some offshore areas have been heavily contaminated with metals as a result of wastewater discharge (Yang et al., 2021). Like other similar metals, Cd remains in the environment indefinitely due to its non-degradability. On the other hand, the nature of nature itself can lead to the coexistence of several pollutants rather than a single pollution (Sun et al., 2024a). For example, emamectin benzoate, a widely used pesticide that has been reported to induce midgut inflammation in carp in combination with microplastics (Shi et al., 2024). Due to the large surface area and certain affinity of microplastics, they can be used as carriers of other pollutants (Zhou et al., 2020). So far, the presence of metals has been detected in microplastics found in several countries around the world, such as offshore China (Wang and Wang, 2018). These literatures have proved that microplastics have the effect of adsorbing metals, but it should be noted that the adsorption level of microplastics is related to the particle size, material and charge of the metal (Godoy et al., 2019; Zou et al., 2020). In addition, microplastics can improve the bioavailability of pollutants after co-treatment with other environmental pollutants, and their toxic effects are greater than those caused by the action of a single poison (Zou et al., 2022). Therefore, when exposed to microplastics and Cd at the same time, earthworms, plankton, and other organisms may be affected by a higher than the effect of a single poison (Chen et al., 2022). As the world's largest duck producer, China accounts for 3/4 of the world's duck breeding (Qin et al., 2023). As a type of waterfowl, ducks live in rivers and pools, feeding on small fish, shrimp or mayflies. In contrast, ducks are more exposed to Cd and microplastics in water than other land poultry. As early as 2021, it was reported that the presence of microplastics was found in the intestines of farmed ducks (Susanti et al., 2021). However, the effect of simultaneous Cd and PVC-MPs exposure on duck kidney has not been reported, therefore, it is necessary to explore the toxic effects of Cd and PVC-MPs on duck kidney.

## Materials and methods

### Chemicals and antibodies

Cadmium chloride **(CdCl_2_)** was purchased from Sigma-Aldrich (St. Louis, MO, USA). PVC microplastics were purchased from the Basesula Chromatography Technology Development Center (Tianjin, China). Hoechst 33258 and hematoxylin and eosin staining kits were purchased from Beyotime (Shanghai, China). Malondialdehyde **(MDA)** assay kit, Total Superoxide Dismutase **(T-SOD)** assay kit (Hydroxylamine method), reduced glutathione **(GSH),** catalase **(CAT),** Total antioxidant capacity assay kit **(T-AOC)** were obtained from Nanjing Jiancheng Bioengineering Institute, Nanjing, China. Anti-Bcl-2, Anti-Drp1, Anti-Nrf1, Anti-PGC-1α, Anti-Cytc, Anti-Fis1 were purchased from Proteintech (Wuhan, China). Anti-β-actin antibodies were purchased from Cell Signaling Technology (Danvers, MA, USA). Anti-SOD_2_, Anti-NQO1, Anti-HO-1, Anti-Nrf2 Abclonal Technology (Woburn,MA,USA). Anti-AC-SOD_2_, Anti-Bax, Anti-Caspase-3, Anti-Caspase-9, Anti-Keap-1, were purchased from Abmart (Shanghai, China).

### Animal experiments

Microplastic concentrations detected in the middle and lower reaches of the Yangtze River in China are around 1 mg/L and well above 10 mg/L in high-exposure areas (Yin et al., 2023). Therefore, the concentration of PVC-MPs used in this study was 10 mg/L. Based on a literature report and our previous research, the exposure concentration of Cd was set at 50 mg/kg (Sun et al., 2023). A total of twenty-eight, five-day-old Muscovy ducks were obtained from a commercial hatchery, and divided into four dietary groups with seven replicates following a completely randomized design: control/untreated group (control, basal diet + freshwater), single Cd exposure group (Cd, basal diet +50 mg/kg Cd in diet + freshwater), single PVC-MPs exposure group (PVC-MPs, basal diet +10 mg/L PVC-MPs in water), and Cd and PVC-MPs co-exposure group (PVC-MPs + Cd, basal diet +50 mg/kg Cd in diet +10mg/L PVC-MPs in water). All ducks were housed in environmentally controlled cages and fed a commercial diet ad libitum for 60 days. The composition and nutrient levels of the basal diet were formulated to meet or exceed the requirements of the National Research Council **(NRC, 1994)** during the starter (1–21 days) and grower (22–60 days) periods. All procedures were approved by the Yangzhou University Animal Care and Use Committee (approval ID: SYXK (Su) 2021- 0027), and all husbandry practices and euthanasia were performed with full consideration of animal welfare.

### Scanning electron microscopy

First of all, the purchased microplastic samples were placed on the shaker and shaken for 6 hours. 1.5-3ml was centrifuged at 12000 g for 30 minutes, then PVC-MPs was dried into powder in an oven at 65 °C and sealed with sealing film to prevent moisture from entering. The morphology of MP was observed on SEM (GeminiSEM 300, Germany).

### Hematoxylin-eosin staining

From each duck, the same portion of the kidney was surgically removed, trimmed, added to a fixative solution (4% para formaldehyde), and fixed for 24 h. The fixative was discarded and the tissue was washed, then dehydrated through an ethanol concentration gradient, made transparent using a xylene solution, and impregnated with wax. Tissue was sectioned using a microtome and attached to a slide. For hematoxylin–eosin staining, the sections were stained with hematoxylin for 7 mins, rinsed Three times with running water (35 seconds each), stained with eosin staining solution for 1 min and 30 seconds, rinsed twice with running water (15 seconds each), and air-dried for microscopic examination. The method of hematoxylin and eosin staining is described in previous studies (Sun et al., 2024a).

### Transmission electron microscopy

The kidney was washed 4-5 times with PBS, fixed in fresh 2.5% glutaraldehyde at room temperature for 2.5 h, and finally transferred to 4 °C for 12 h. After 1 h incubations in 0.1 M sodium cacodylate buffer, they were post-fixed in 1% v/v osmium tetroxide in cacodylate buffer for 2.5 h in darkness. Subsequently, they were dehydrated in acetone, using 15 mins changes at 10%, 20%, 30%, 50%, 70%, and 95% v/v, and three times in 100% acetone. To prepare the samples, we used Spurr's resin in acetone and underwent a series of 15 mins changes at 25%, 50%, 75% v/v, and three times in 100% Spurr's resin. Afterward, we polymerised the samples 12 h in Spurr's resin at 60°C. We made semi-thin sections of 300 nm thickness using an ultramicrotome (UC6; Leica Microsystems, Vienna) equipped with a diamond ultra-knife (DiATOME). After producing 80 nm ultrathin sections and collecting them on copper slot grids, we mounted semi-thin sections in Congo red and viewed them using an epifluorescence microscope equipped with a TRITC filter. The ultra-thin sections were then stained for 35 mins in 1% uranyl acetate at 37°C and for 15 mins in 3% lead citrate at 20°C. Finally, we examined the grids using a transmission electron microscope (HT7800, HITACHI, Japan).

### Detection of oxidative stress-related indexes

First, based on the results of the preliminary experiment, a certain amount of tissue was weighed to prepare a 5 or 10% tissue suspension. Centrifuge at 3000 × g for 7 mins, and measure the supernatant. The cellular activities of MDA, T-SOD, CAT, T-AOC, and GSH were measured using commercial kits from the Nanjing Jiancheng Bioengineering Institute (Nanjing, China) according to the manufacturer's instructions.

### Immunohistochemistry

Immunohistochemistry of HO-1, Nrf1, Caspase-3 and Caspase-9 was performed with reference to previous studies (Wang et al., 2023). The slices from Kidney were incubated in oven at 60°C for 45 mins, and then dewaxed in dimethylbenzene for 2 times, further hydration using the correspondingly ethanol solution at different concentrations (70% to 100%). Next, the slices were incubated with 1% hydrogen peroxide **(H_2_O_2_)** for 15 min at room temperature (25°C). The slices were incubated with primary antibodies against the HO-1 and Nrf1, Caspase-3 and Caspase-9 at 4°C overnight. After the end of incubation, secondary antibodies were incubated for 30 min at 37°C. Finally, the antigen-antibody complexes were obtained by 3,30-diaminobenzidine **(DAB).**

### TUNEL staining

In order to detect the apoptosis of kidney cells, we measured the apoptosis rate of kidney cells according to the method of (Chen et al., 2024). Detection of paraffin-embedded kidney sections fixed with 4% paraformaldehyde by terminal deoxynucleotidyl transferase-mediated dUTP-biotin nick end labeling (TUNEL) (Beyotime, China) according to the manufacturer's instructions.

### Western blotting analysis

For samples such as tissues, a certain amount of tissue was weighed and normal saline was added to make a 10% tissue homogenate centrifuged, lysed in Radioimmunoprecipitation assay **(RIPA)** Lysis Buffer at 4°C for 20 mins, and treated with ultrasound twice for 5 s each time. The samples were centrifuged at 12,000 × g for 12 mins, the supernatant was collected, and the protein concentration in the supernatant was determined using a bicinchoninic acid **(BCA)** kit (Yeasen BioTechnology). The different samples were adjusted to the same protein concentration with loading buffer, and placed at 100°C for 10 mins. Proteins were separated using sodium dodecyl sulfate-polyacrylamide gel electrophoresis and transferred to polyvinylidene difluoride membranes (Millipore, Billerica, MA, USA). The membranes were subjected to blocking, washing with Tris Buffered Saline with Tween® 20 **(TBST),** primary antibody incubation, and secondary antibody incubation, after which the densities of the immunoreactive protein bands were measured using ImageJ Laboratory software (NIH, Bethesda, MD, USA).

### Quantitative real-time polymerase chain reaction **(qRT-PCR)**

Total RNA of kidney samples were extracted using TRizol reagent for solubilization and purification (Thermo Fisher Scientific Inc.; Waltham, MA) as previously reported (Wang et al., 2024). Pure RNA was reverse transcribed to cDNA using a HiScript Q RT SuperMix for qPCR (+gDNA wiper) kit (Nanjing Vazyme Biotech Co., Ltd.; Nanjing, Jiangsu, China). Primer sequences were used to qRT-PCR (Table 1). The cDNA templates were amplified using a Hieff qPCR SYBR Green Master Mix kit (Yeasen Biotechnology Co., Ltd.; Shanghai, China). The reaction methods were as follows: 95°C for 30 s; 40 cycles of 95°C for 5 s, 60°C for 34 s; and 60°C for 15 s. The mRNA expression of the target genes was calculated using the comparative threshold cycle method (2^-ΔΔCt^) method, and β-actin was correspondingly used as the housekeeping gene for normalization.

**Table 1:** The primer sequences were used for qRT-PCR.

Gene name	Primer sequences (5−3′)	Length(bp)
Cytc	Forward:CTTCTCTGCCTACCGTCGTC Reverse:AAGTGGAGCAGGACCTTTCC	40
Caspase-3	Forward:TTGTCAGCCTCGCAGTTGA Reverse:CCCGAACGAGATGACAGTCC	39
Caspase-9	Forward:CCTGTGGTGGAGACCAGAAA Reverse:AGCAGGTCTTCAGAATGGGC	40
Bax	Forward:CAGTGAGGAGCGTTCGAGG Reverse:CAGCAGGCTGAACTGGAAGA	39
Bcl-2	Forward:GTGGCCTTCTTCGAGTTCGG Reverse:CAGTCAGAACTGATCCAGCCT	40
β-actin	Forward:CAGCACGATGAAAATCAAGATCA Reverse:CAAGGGTGTGGGTGTTGGTAA	44

### Statistical analysis

All experimental data were analyzed using one-way analysis of variance **(ANOVA)** with SPSS 27.0 (IBM SPSS Inc., Chicago, IL). Quantitative data are presented as means ± SD. Significance was indicated by *p* < 0.05, and high significance by *p* < 0.01, while P > 0.05 was considered not significant (ns). All experiments were performed in triplicate.

## Results

### Exposure to Cd and PVC-MPs induce kidney damage

**Fig. 1:**
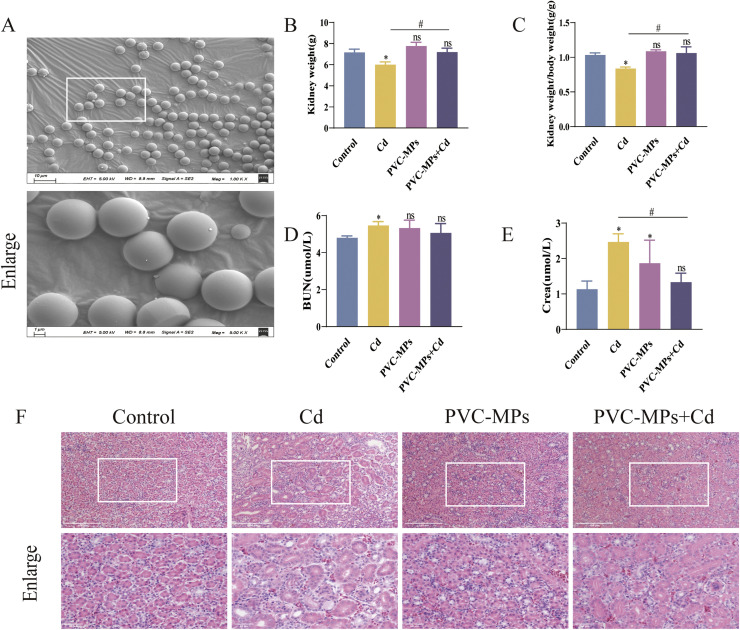
Exposure to Cd and PVC-MPs induces kidney damage. (A): Scanning electron microscopy observations of PVC-MPs morphology, scale bars = 10 µm, Enlarge scale bars = 1 µm. (B): Kidney weight. (C): Kidney weight/Body weight. (D):blood urea nitrogen. (E): Crea. (F): Histopathology of Kidney observed using HE staining, scale bars = 100 µm, Enlarge scale bars = 50 µm. Results are shown as the mean ± SD (*n* = 3). Compared with the Control group, ns, not significant, *p < 0.05, **p < 0.01. Compared with the PVC-MPs+Cd group, ^#^*p* < 0.05, ^##^*p* < 0.01.

First, we observed the characterization of PVC-MPs using scanning electron microscopy, as shown in (Fig. 1A), the morphology of PVC-MPs is regularly oval particles of uniform size. Next, we measured the weight and organ index of the kidney, as shown in (Fig. 1B-C), compared with the control group, the application of Cd affected the development of the kidney, reducing kidney weight, while the PVC-MPs caused slight swelling of the kidney, and there was no significant change in the kidney after PVC-MPs+Cd treatment. In addition, compared with the control group, the contents of urea nitrogen and creatinine increased in the Cd and PVC-MPs groups, but there was no significant change in urea nitrogen and creatinine contents in the PVC-MPs+Cd treatment group (Fig. 1D-E). HE staining was used to observe the histopathological changes in the kidney tissue, and the results showed that compared with the control group, the Cd group had a disordered tubular arrangement, vacuolar degeneration, different sizes, and glomerular swelling. In the PVC-MPs group, glomerular atrophy was atrophied, and the tubular staining became pale and the space was blurred. After PVC-MPs+Cd treatment, the renal tubules and glomeruli were slightly swollen, the staining became lighter, and the size of the nucleus was different (Fig. 1F).

### Effects of Cd and PVC-MPs on oxidative stress in kidney

**Fig. 2:**
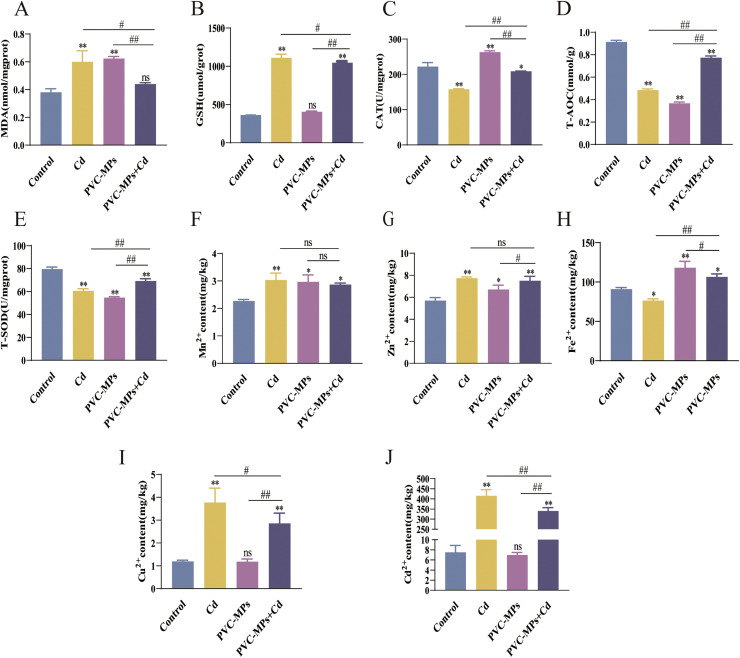
Effects of Cd and PVC-MPs on oxidative stress in kidney. (A-E): The oxidase levels of MDA in kidney tissues of different groups were measured, as well as the antioxidant enzyme levels of GSH, CAT, T-AOC, and T-SOD. (F-J): Mn, Zn, Fe, Cu, Cd contents in dried kidney tissue were determined through flame atomic absorption spectroscopy. Results are shown as the mean ± SD (n=3). Compared with the Control group, ns, not significant, *p < 0.05, **p < 0.01. Compared with the PVC-MPs+Cd group, ^#^*p* < 0.05, ^##^*p* < 0.01.

In order to verify the effect of Cd and PVC-MPs alone or in combination on Kidney oxidative stress, the activity of kidney-related antioxidant enzymes was detected, and the results were shown in (Fig. 2A-E). Compared with the control group, the Cd and PVC-MPs alone treatment increased the MDA content and decreased the activity of T-AOC and T-SOD, and Cd increased the GSH content and decreased the CAT activity. Interestingly, there was no change in GSH content and increased CAT activity in the PVC-MPs alone treatment group. There was no change in MDA content in PVC-MPs+Cd treatment group, but the content of GSH increased, and the activities of T-AOC and T-SOD decreased. Immediately afterwards, we measured the trace element levels. Compared with the control group, the Cd and PVC-MPs alone treatment increased the Mn^2+^ and Zn^2+^ contents, and the Cd group decreased the Fe^2+^ content and increased the Cu^2+^ content. However, the Fe^2+^ content was increased, but the Cu content did not change in the PVC-MPs group. Meanwhile, the contents of Mn^2+^, Zn^2+^, Fe^2+^, Cu^2+^, Mn^2+^, Fe^2+^ and Cu^2+^ increased in PVC-MPs+Cd group (Fig. 2F-I). Finally, we measured the Cd content in the kidneys, and the results showed that there was an increased Cd content in the Cd group compared to the control group, no change in Cd content in the PVC-MPs group, and a sharp decreased Cd content in the PVC-MPs+Cd treatment group compared to the Cd alone treatment group (Fig. 2J).

### Cd and PVC-MPs induce mitochondrial damage

**Fig. 3:**
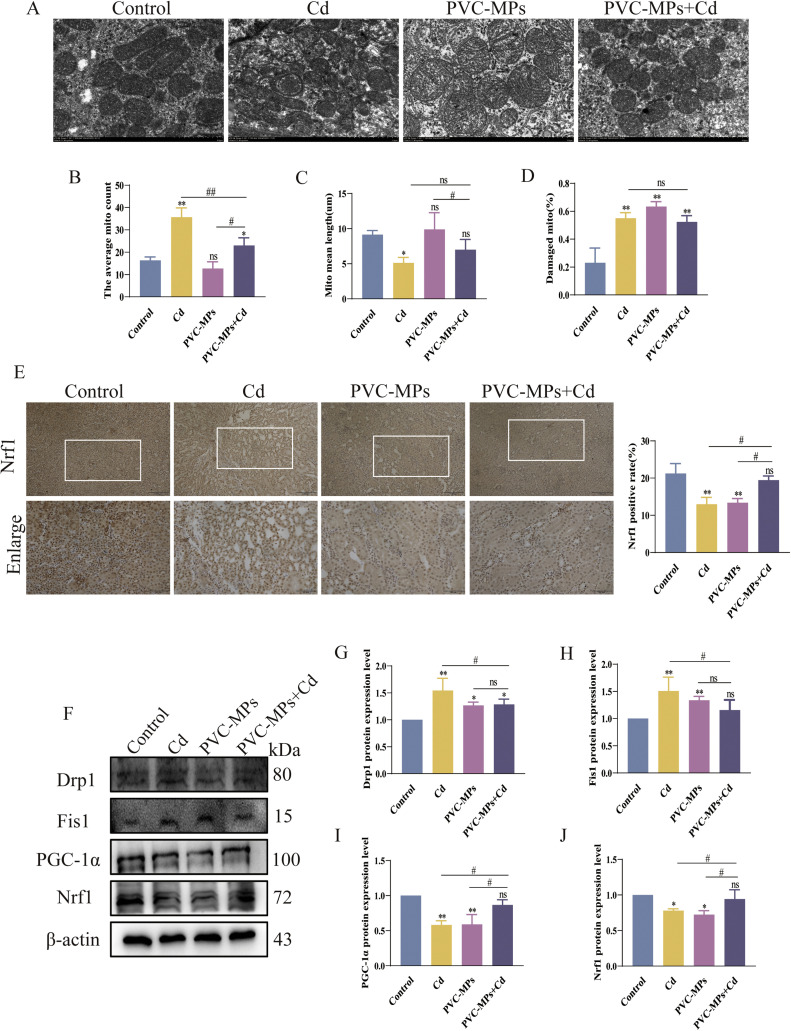
Cd and PVC-MPs induce mitochondrial damage. (A): Transmission electron microscopic observation of mitochondria microstructure, scale bars = 1 µm. (B): The average mitochondrial count. (C): mitochondrial mean length. (D): Damaged mitochondrial. (E): The distribution of Nrf1 in Kidney were observed by immunohistochemistry. scale bars = 100 µm, Enlarge scale bars (down) = 50 µm. (F-J): The expression of Drp1, Fis1, PGC-1α and Nrf1 were detected by western blot. Results are shown as the mean ± SD (*n* = 3). Compared with the Control group, ns, not significant, *p < 0.05, ^⁎⁎^*p* < 0.01. Compared with the PVC-MPs+Cd group, ^#^*p* < 0.05, ^##^*p* < 0.01.

The ultrastructure of kidney mitochondria was observed by transmission electron microscope. The results showed that the mitochondrial outer membrane was intact and clear in the control group. But, the number of mitochondria increased, the mitochondrial length decreased, the number of damaged mitochondria increased, and the mean length decreased in the Cd treatment group, while the mitochondria in the PVC-MPs group were swollen, the internal crest was broken, and the proportion of damaged mitochondria increased. Interestingly, the mitochondrial damage after PVC-MPs+Cd treatment was similar to that treated with Cd alone, with an increase in the number of mitochondria, a decrease in average length and an increase in the proportion of mitochondrial damage (Fig. 3A-D). Similarly, immunohistochemistry was used to verify the expression of Nrf1 protein, and the expression of Nrf1 protein was reduced in Cd and PVC-MPs alone treatment groups, and there was no significant change in Nrf1 expression after PVC-MPs+Cd treatment (Fig. 3E). Next, we further examined the mitochondrial function-related proteins. WB results showed that the protein expression of Drp1 and Fis1 increased, and the expression of PGC-1α and Nrf1 decreased in the Cd and PVC-MPs alone treatment groups, while the protein expression of Drp1 increased after PVC-MPs+Cd treatment, and there was no significant change in Fis1, PGC-1α and Nrf1 proteins (Fig. 3F-J).

### Effects of Cd and PVC-MPs on Nrf2 signaling pathway

**Fig. 4:**
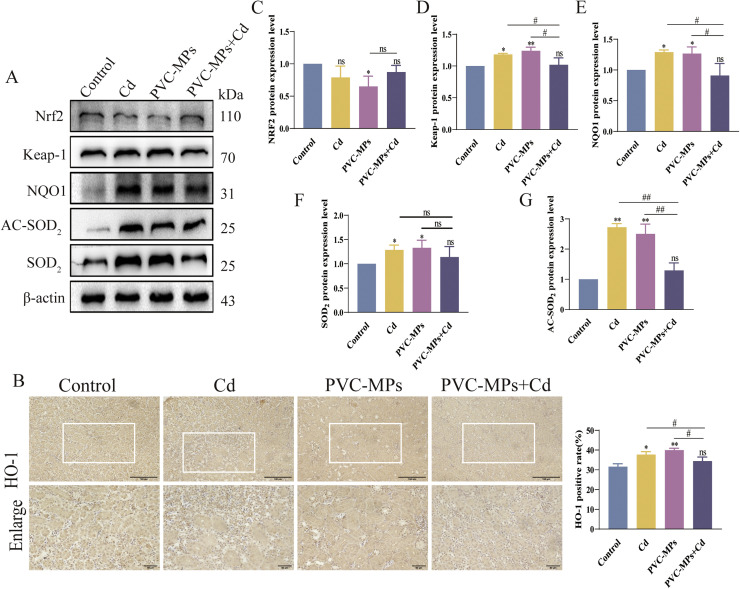
Effects of Cd and PVC-MPs on Nrf2 signaling pathway. (A): The expression of Nrf2, Keap-1, NQO1, AC-SOD_2_ and SOD_2_, were detected by western blot. (B): The distribution of HO-1 in Kidney were observed by immunohistochemistry. scale bars =100 µm, Enlarge scale bars = 50 µm. Results are shown as the mean ± SD (*n* = 3). (C-G): Expression of Nrf2, Keap-1, NQO1, AC-SOD_2_, and SOD_2_-related proteins. Compared with the Control group, ns, not significant, *p < 0.05, ^⁎⁎^*p* < 0.01. Compared with the PVC-MPs+Cd group, ^#^*p* < 0.05, ^##^*p* < 0.01.

Nrf2 is a ubiquitously expressed transcription factor that regulates the levels of antioxidant and detoxification enzymes, such as HO-1 and NQO1. WB and immunohistochemistry were performed to verify the expression of proteins related to Nrf2 signaling pathway. As shown in (Fig. 4A-G), WB results showed that Cd and PVC-MPs alone decreased the expression of Nrf2 protein and increased the expression of Keap-1, NQO1, SOD_2_ and SOD_2_ precursor protein, but there was no significant change after PVC-MPs+Cd treatment. In addition, the immunohistochemistry results showed that compared with the control group, the expression of HO-1 protein was increased by Cd and PVC-MPs alone, while the expression of HO-1 protein was not significantly changed after PVC-MPs+Cd treatment.

### Cd and PVC-MPs induce kidney apoptosis

**Fig. 5:**
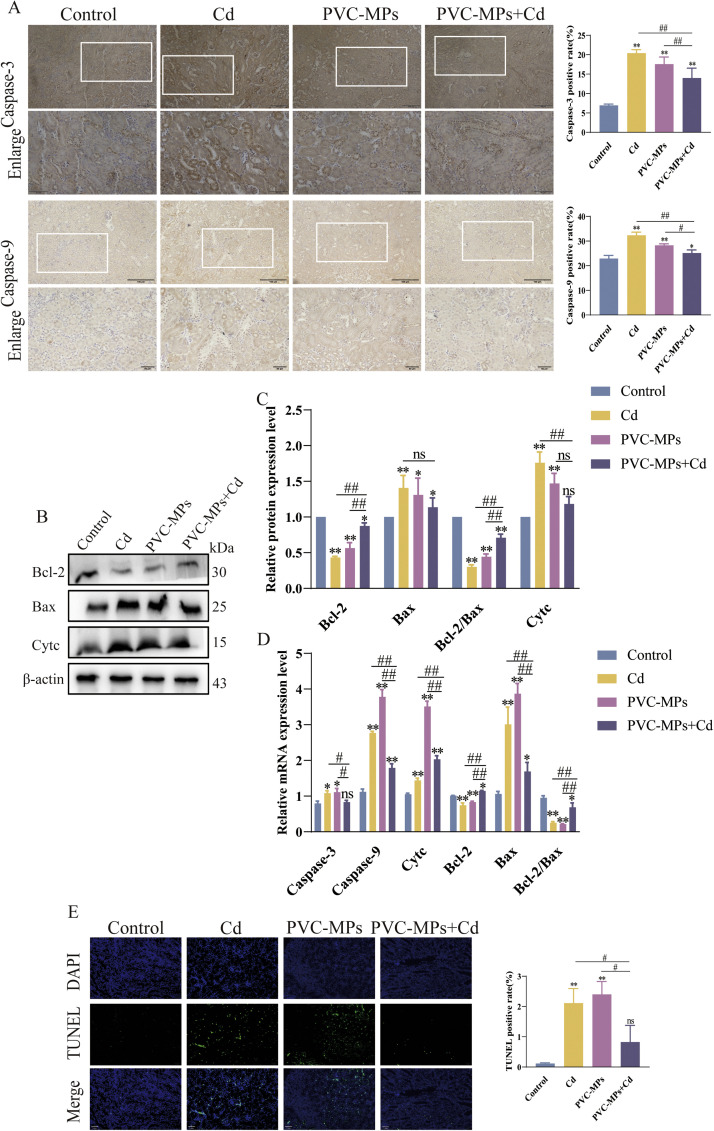
Effects of Cd and PVC-MPs on Nrf2 signaling pathway. (A): The distribution of Caspase-3 and Caspase-9 in Kidney were observed by immunohistochemistry. scale bars =100 µm, Enlarge scale bars = 50 µm. (B-C): The expression of Bcl-2, Bax and Cytc, were detected by western blot. (D): qRT-PCR analysis of Apoptosis related Caspase-3, Caspase-9, Cytc, Bcl-2 and Bax. (E): TUNEL staining column analysis diagram. Results are shown as the mean ± SD (*n* = 3). Compared with the Control group, ns, not significant, *p < 0.05, **p < 0.01. Compared with the PVC-MPs+Cd group, ^#^*p* < 0.05, ^##^*p* < 0.01.

First of all, the expression of Caspase-3 and Caspase-9 protein was verified by immunohistochemistry. Compared with the control group, the expression of Caspase-3 and Caspase-9 increased after treatment with Cd and PVC-MPs alone or in combination (Fig. 5A). The WB results showed that compared with the control group, the expression of Bcl-2 protein and the ratio of Bcl-2/Bax decreased, and the expression of Bax and Cytc proteins increased after Cd and PVC-MPs alone treatment, and the Bcl-2 protein and Bcl-2/Bax ratios of PVC-MPs+Cd treatment were restored compared with the PVC-MPs and Cd alone treatment, and the expression of Bax and Cytc proteins was similar to that of the control group (Fig. 5B-C). In addition, we further validated the mRNA expression of Caspase-3, Caspase-9, Cytc, Bcl-2, and Bax, when treated with Cd and PVC-MPs alone, the expression levels of Caspase-3, Caspase-9, Cytc and Bax mRNA increased, while the mRNA expression level of Bcl-2 and the ratio of Bcl-2/Bax decreased. There was no significant change in Caspase-3 after PVC-MPs+Cd treatment, but the mRNA expression of Caspase-9, Cytc, Bcl-2 and Bax increased and the ratio of Bcl-2/Bax decreased (Fig. 5D). Finally, we measured the apoptosis rate of renal cells by TUNEL. Compared with the control group the positive signal of TUNEL was significantly increased in Cd and PVC-MPs alone group and the apoptosis rate significantly increased. Interestingly the apoptosis rate decreased after PVC-MPs+Cd (Fig. 5E).

## Discussion

Ducks, being waterfowl, live in rivers and are thus at a greater risk of exposure to environmental toxins compared to land-based poultry (Liu et al., 2023). Cd-induces excessive Reactive Oxygen Species (ROS) production, which causes oxidative damage to DNA, which destroys the oxidation / antioxidation balance. (Li et al., 2010; Wei et al., 2015). A large number of studies have shown that oxidative stress is an important mechanism of Cd-induced nephrotoxicity (Nair et al., 2015), Specific biomarkers (MDA, SOD and CAT) are strongly associated with oxidative stress due to Cd exposure (Shagirtha et al., 2011).There are also different data to prove that exposure of early juveniles of Rainbow Angelfish to polystyrene microplastics (0, 50 or 500 mg/L) and Cd (0 or 50 mg/L) has no significant effect on the growth and survival of juveniles (Naqash et al., 2020). In addition, microplastics can act as carriers of other pollutants, but the exposure of microplastics to other pollutants mainly stems from two aspects: the substances added to the plastic during the manufacturing process and the substances in the environment adsorbed by the microplastics themselves (Wang et al., 2022). In our study, lipid peroxidation **(MDA)** levels were exacerbated by Cd or PVC-MPs alone, while there was no significant change in lipid peroxidation levels when Cd was combined with PVC-MPs. Then the levels of GSH, CAT, T-AOC and T-SOD were further measured. The results showed that compared with the control group, Cd or PVC-MPs alone could disturb the normal enzyme level in the kidney, and the enzyme level seemed to recover after combined treatment.

The balance of elemental metabolism is necessary for normal cells to perform biological functions, such as signal transduction or enzyme activity (Zhang et al., 2018). The physicochemical properties of Cd determine its toxic effects, which means that trace elements with similar chemical properties can interact with Cd, and this interaction can occur at different stages of the absorption, distribution and excretion of trace elements (Jemai et al., 2007). For example, CAT and SOD enzyme activities are regulated by metal elements such as Fe, Mn, and Zn (Sun et al., 2024a), while exposure to microplastics or Cd may affect the elemental metabolism of normal cells. The results of this study showed that exposure to Cd or PVC-MPs could disrupt the normal trace element metabolism of Mn^2+^, Zn^2+^, Fe^2+^ and Cu^2+^. Interestingly, PVC-MPs did not affect the normal metabolism of Cu^2+^. In addition, the deposition of Cd^2+^ in the kidney was significantly lower after the combined treatment of Cd and PVC-MPs than that in the Cd treatment alone. Based on the results of trace element changes, this may explain the changes in antioxidant enzymes to some extent.

Mitochondrial fission provides quality control by isolating damaged or dysfunctional components from a healthy mitochondrial network (Meyer et al., 2017). Conversely, mitochondrial fusion is thought to rescue damaged mitochondria by rapidly balancing mtDNA copies, matrix, and membrane components, and is therefore essential for maintaining mitochondrial function (Abrisch et al., 2020). Drp1 is a cytoplasmic protein that binds to Fis1 on the surface of mitochondria and causes division (Dong et al., 2022). There is evidence that DRP1-dependent mitochondrial fission leads to Cd -induced hepatotoxicity (Xu et al., 2013). Overexpression of Fis1 and Drp1 not only promotes mitochondrial hyperfission but also promotes cell metastasis of hepatocellular carcinoma cells (Yu et al., 2021). Previous studies have shown that reduced PGC-1α and TFAM expression leads to impaired mitochondrial biogenesis and a decrease in the number of mitochondria, leading to mitochondrial biogenesis-mediated neurotoxicity (Goyal et al., 2021). Other literature has also shown that increasing PGC-1α and Nrf1 expression can improve mitochondrial function and glucose uptake in skeletal muscle in diabetic mice (Meng et al., 2020). In addition, PGC-1 α binds to other transcription factors (such as Nrf1 or Nrf2) to promote fatty acid oxidation and biogenesis in cardiac mitochondria, which is important for the maintenance of cardiac function (Dorn et al., 2015; Scarpulla et al., 2012). In our study, we found that Cd and PVC-MPs significantly up-regulated Fis1 and Drp1, and down-regulated the expression of PGC-1α and Nrf1, which was further confirmed by immunohistochemistry. Our results demonstrate that under the influence of Cd and PVC-MPs, the balance of mitochondrial division and fusion is disturbed, while PGC-1α, the main regulator of mitochondrial biological function, and its downstream proteins are inhibited, the normal biological function of mitochondria is disrupted, and mitochondrial homeostasis is disrupted, thereby further inducing cell damage.

The toxicity of microplastics and Cd is often linked to oxidative stress, and studies have shown that ingestion of Cd increases free radicals in cells (Yee et al., 2021). Nrf2 signaling mitigates Cd-induced multi-organ toxicity, including in the liver (Mohajeri et al., 2017) and kidney (Campbell and Heikkila, 2018),Normally present in the cytoplasm as a heterodimer of Keap1-Nrf2, once the cell is stimulated by ROS, Nrf2 is released and rapidly transferred to the nucleus, initiating the transcriptional expression of downstream target genes (HO-1 and NQO1)(Baird and Yamamoto, 2020). HO-1 is the most actively expressed subtype of heme oxygenase **(HO)** (Sadasivam et al., 2022), a powerful antioxidant that protects cells from ROS-mediated damage such as DNA damage (Shaw and Chattopadhyay, 2020). NQO1 is an important detoxification enzyme for maintaining cellular redox stability by maintaining the reduced form of cellular antioxidants and removing superoxide (Jeddi et al., 2017; Siegel et al., 2004). Similarly, SOD_2_ is considered a scavenging enzyme whose activity is related to the mitochondrial level (Zeng et al., 2014). Therefore, SOD_2_ activity is essential for maintaining mitochondria-derived reactive oxygen species homeostasis (Zhu et al., 2012). The results showed that the Nrf2 protein decreased and the expression of Keap-1 increased after Cd or PVC-MPs alone, suggesting that Nrf2 protein entered the nucleus and increased the protein expression of HO-1, NQO1, SOD_2_ and SOD_2_ precursors in the kidney, and the expression of HO-1 was verified by immunohistochemistry, which was consistent with the changes of other related downstream proteins. However, there was no significant change in Nrf2 and downstream target proteins after PVC-MPs+Cd treatment. Therefore, according to our findings, treatment of Cd or PVC-MPs alone can induce the activation of the Nrf2 pathway, however, the combined treatment of PVC-MPs+Cd has no significant effect on the Nrf2 pathway, which may be due to the reduction of Cd accumulation after the combined treatment of PVC-MPs+Cd.

Life depends on a delicate balance between anabolism (synthesis) and catabolism (degradation), apoptosis (also known as type I cell death) is a well-studied example of programmed cell death (Prerna and Dubey, 2022). Apoptosis is a complex phenomenon that includes both external and internal pathways (Souza-Arroyo, Fabián, Bucio-Ortiz, Miranda-Labra, Gomez-Quiroz and Gutiérrez-Ruiz, 2022), It plays an important role in vertebrate development, cellular homeostasis, and tumor suppression (Ke et al., 2018). Many studies have demonstrated that the apoptosis signaling pathway is mediated by mitochondria (Yang et al., 2019),under the regulation of Bcl-2 family proteins, mitochondrial membrane permeability changes, and cytochrome C (Cytc) is released from mitochondria into the cytoplasm (Kaushik et al., 2021). For example, recent studies have confirmed that BPA and low selenium can induce mitochondrial damage and apoptosis in chicken pancreatic cells (Sun et al., 2024b). Much has been written about Cd and microplastics inducing apoptosis. Sheng et al. found that PS-MPs and/or Cd activate the hepatic antioxidant pathway Nrf2-Keap1 and affect the endogenous apoptotic signaling pathway P53-Bcl-2/Bax, thereby promoting hepatocyte apoptosis (Sheng et al., 2024). In addition, Zhang et al. found that 100 nm PS microplastic particles caused more severe oxidative damage and apoptosis than 1 μm PS, and similarly, 100 nm PS+Cd+1umPS had the most severe damage to kidney function, pathological changes and apoptosis (Zhang et al., 2024). In this study, PCR results showed that the ratios of Caspase-3, Caspase-9, Cytc, Bcl-2, Bax, and Bcl-2/Bax were consistent with changes in tissue proteins induced by Cd and PVC-MPs alone, while the ratio of Bcl-2 to Bcl-2/Bax was reduced. In addition, TUNEL staining results showed that the use of Cd and PVC-MPs alone increased the rate of renal apoptosis, and the rate of renal apoptosis was reduced after PVC-MPs+Cd treatment compared with Cd and PVC-MPs alone. These findings suggest that mitochondrial pathway-mediated apoptosis plays a role in Cd versus PVC-MPs-induced kidney injury.

## Funding

This work was supported by the National Key R&D Program of China (No. 2023YFD1801100), the National Natural Science Foundation of China (Nos. 32072933, and 32273086), 111 Project D18007, a Project Funded by Priority Academic Program Development of Jiangsu Higher Education Institutions (PAPD), and the Qing Lan project of Yangzhou University.

## Declarations

### Ethics approval and consent to participate

Every procedure and protocol involving animals were permitted by the Yangzhou University Comparative Medical Center (Jiangsu Province, China). The study was based on the Guide to moral Control and Supervision in Animal Conservation and use.

## Consent for publication

Not applicable

## Availability of data and material

The datasets used and/or analyzed during the current study are available from the corresponding author on reasonable request.

## CRediT authorship contribution statement

**Hui Zou:** Conceptualization, Writing – original draft, Writing – review & editing. **Jie Song:** Conceptualization, Writing – original draft. **Xianzu Luo:** Conceptualization, Writing – original draft. **Waseem Ali:** Software. **Sifan Li:** Data curation. **Ling Xiong:** Data curation. **Yan Chen:** Data curation, Software. **Yan Yuan:** Data curation. **Yonggang Ma:** Visualization, Investigation. **Xishuai Tong:** Visualization, Investigation. **Zongping Liu:** Writing – review & editing.

## Declaration of competing interest

The authors declare that they have no known competing financial interests or personal relationships that could have appeared to influence the work reported in this paper.
